# Understanding Neural Oscillations in the Human Brain: From Movement to Consciousness and Vice Versa

**DOI:** 10.3389/fpsyg.2019.01930

**Published:** 2019-08-27

**Authors:** Ana Maria Cebolla, Guy Cheron

**Affiliations:** ^1^Laboratory of Neurophysiology and Movement Biomechanics, Université Libre de Bruxelles, Brussels, Belgium; ^2^Laboratory of Electrophysiology, Université de Mons-Hainaut, Mons, Belgium

**Keywords:** oscillation, movement, consciousness, brain, intention

## Introduction

Considering perinatal human maturation of the cerebral cortex, movement occurs before consciousness. Considering human motor control, consciousness endorses voluntary action. On the arduous road to understanding consciousness and its mechanisms, new and refined experimental paradigms may determine the next avenue. From this perspective, involuntary and voluntary movements can be considered the starting point of consciousness, the consequence of consciousness, or just a tool for approaching it. From the recent consciousness models including neural group selection and the integrated information theories, we highlight some of the experimental considerations that could indicate that movement frames consciousness. On the one hand, it may be a trigger for searching the key features in the environment (e.g., eye-scan path), but on the other hand it may conclude the final reporting of consciousness (e.g., goal vocal or manual-oriented action). The aim of this opinion paper is to encourage a discussion in the framework of the research topic, “Understanding Neural Oscillation in the Human Brain: Consciousness of Movement Execution,” and to promote new scientific interest about the hypothesis that movement is inescapable in understanding consciousness. In the following subsections we underline that oscillatory brain mechanisms integrate movement into the dynamics of the default mode network, the bottom up and top down modulations, the intentional actions in social contexts, the individual selfness and body identity, suggesting that movement may be essential to consciousness and that the oscillations-movement-consciousness triad should be inextricable.

Before the emergence of long-range cortical connections that allow consciousness (Varela et al., 2001), the early emergence of spontaneous electrical activity in the brain is based on well-shaped intermittent spontaneous oscillations that produce fetal movements (Khazipov et al., 2004; Khazipov and Milh, 2018). In rat pups, these stochastic motor actions, which are described as “popcorn” movements, generate reafferentation activities via the thalamocortical system, allowing self-organized dynamics in the brain (Buzsaki, 2011). These immature movements allow exploration of the physic world, and finally conduct humans toward self-consciousness, for which body perception and action play an important role in elaborating a sense of self and differentiating between self and others (Keromnes et al., 2018).

Recent theories about consciousness (Edelman, 2003; Seth et al., 2006; Edelman et al., 2011; Park and Blanke, 2019) have paved the way for new experimental paradigms. Thirteen features have been proposed (Seth et al., 2006) in order to better characterize the theoretical frame of reference for consciousness. Among these items, the first three established that: (1) fast, irregular, and low-amplitude oscillations (~12–70 Hz) convey consciousness; (2) these oscillatory neuronal activities are organized by the thalamocortical system acting as a “dynamic core” modulated by subcortical influences; and (3) consciousness is dispatched in different cortical areas depending on the conscious content. The other 10 items highlight that the conscious events are unitary, and that only one conscious experience emerges at a time. Accordingly, the theory of neuronal group selection (TNGS) (Edelman, 1987) is advanced as a biological foundation of consciousness. Following the TNGS, Darwinistic selection has ontogenetically shaped neuronal circuits based on positive or negative outcomes on the environment and related feedback. In this context, the reentry process linking numerous brainstem nuclei with the thalamocortical system (Edelman and Gally, 2013) and the recurrent circuit in the cortex that assumes the function of working memory (McCormick, 2001) are crucial for consciousness (Edelman et al., 2011). This implies that consciousness is a dynamic embodied process (Seth et al., 2006) that is closely related not only to voluntary movement production, but also to internal body signals from visceral organs (Park et al., 2018; Park and Blanke, 2019). Electroencephalography (EEG) (Haegens et al., 2010; Braboszcz and Delorme, 2011; Baird et al., 2014; Horschig et al., 2014; Shafto and Pitts, 2015; Koivisto et al., 2016; Ye et al., 2019) and functional magnetic resonance imaging (fMRI) (Kucyi, 2018; Kucyi et al., 2018; Demertzi et al., 2019; Golkowski et al., 2019; Liégeois et al., 2019; Yin et al., 2019) are commonly used to study general attention and consciousness in humans. Although the high temporal resolution of the EEG (timing in milliseconds range) and high spatial resolution of the fMRI (location in millimeters range) are viewed as complementary for understanding neural processes (Bréchet et al., 2019; Shen et al., 2019), recent evidence (Itthipuripat et al., 2019) demonstrated that hemodynamic attentional modulations measured in the early sensory cortex are differentially related to evoked EEG potentials, as they are linked more to later than early evoked potentials.

## The Basic Control of the Default Mode

A first trivial observation is that experiences related to consciousness involve a basic awareness state, during which the participant can verbally report self-consciousness. This implies that any kind of experimental tentative to detect the emergence of consciousness related to a sensory item or the production of a free will action depends on the quality of the reference state, commonly considered as the resting or default mode network (DMN) (Raichle et al., 2001; Raichle, 2015), which presumes a non-relevant task state. A significant correlation between the global field alpha power and respiration was demonstrated in the eyes-closed resting state (Yuan et al., 2013). Other internal (visceral) movements, such as heartbeats, contribute not only to the DMN state, but also to encoding the self (Babo-Rebelo et al., 2016, 2019; Azzalini et al., 2019). The relative permanency of the self-referential and time to time modulation of mind wandering (Smallwood and Schooler, 2015; Kucyi, 2018) should be taken into account as a basic experimental control for the measurement of conscious events (Northoff et al., 2010). Until now, such control remains difficult to generalize. Recently, Davey et al. (2016) demonstrated that body-self-referential processes are assumed by the posterior cingulate cortex and regulated by the medial prefrontal cortex, which are two areas of the DMN. In addition, slow fluctuations in the position of the eyes during visual fixation influence the intrinsic DMN activity (Fransson et al., 2014). This indicates that both gaze position and body posture likely influence the DMM activity, as illustrated by “zazen” meditation practice (Brandmeyer et al., 2019), during which postural control is required. As wellness behavioral methods emphasize attention on the breathing movements to enhance body consciousness, the related EEG oscillations linked to respiration and cardiac activity should be integrated into protocols.

## The Oscillatory Dialogue Between Bottom-Up and Top-Down

The bottom-up process is recognized as stimulus-driven processing capable of producing movement without volition and being outside the scope of consciousness. In contrast, top-down is considered expectation-driven processing (Engel et al., 2001), which implies voluntary action realized in full consciousness. Three stages of voluntary movement have been differentiated: the first is preconceptual and involves an inner impulse, constituting the bottom-up component of the action; the second is where the intention is conceptual, specific, and more conscious; and the third is where the decision is made whether to perform the action, constituting the top-down component (Schmidt et al., 2016). Neuronal oscillations underlie bottom-up and top-down processes (Engel et al., 2001; Varela et al., 2001) by linking separate and distant brain areas involved at different levels of the network and ensuring complex and integrative functions. The functional integration role of oscillations make them an attractive candidate mechanism for approaching a high level of complexity such as consciousness (Crick, 1984; Tononi and Edelman, 1998). Thus, the dynamics of oscillations could underlie the mechanisms of unity of consciousness (Cleeremans, 2003). In the same way that the 40 Hz oscillation in the visual cortex (Eckhorn et al., 1988; Gray et al., 1989) has been indicate to be the neural correlate of visual perceptive consciousness, the thalamocortical 40 Hz oscillation may play a major role synchronizing the firing of separate and differentiated cortical neural populations underlying motion consciousness (Llinás, 2001). Along these lines, self-consciousness was recently shown to involve gamma oscillations (~40 Hz) carried out by dopamine-dependent recurrent GABAergic neurons located in a cortical network connecting the medial frontal pre-area, anterior cingulate area, medial parietal area, and posterior cingulate area (Lou et al., 2017).

Despite the early scientific and clinical interest of Charcot (1882) and Ramóny Cajal (1889) in hypnosis, its related underlying mechanisms remain unresolved (Sala et al., 2008). Recent attempts have been made to dissociate bottom-up and top-down processing during hypnosis, which would modulate consciousness and allow the discrimination of reports of actual movement from the intention to move (Terhune et al., 2017). Hypnosis has been used as a maneuver to enhance bottom-up processing in responders by reducing the top-down control exerted by the prefrontal cortex (Gruzelier and Warren, 1993). Notably, the motor paralysis induced by hypnosis would not be due to direct motor inhibition, but to complex self-monitoring processes generated by the suggestion guiding feigned behavior (Cojan et al., 2009, 2013). Interestingly, individual differences in hypnotic susceptibility have been supported by different levels of EEG phase synchronization in the frontal lobe (Egner et al., 2005; Baghdadi and Nasrabadi, 2012), suggesting that hypnotic susceptibility is linked to the efficiency of the frontal attention system.

## Intentional Actions in Social Context

The way we process the intentional actions of others in a social context can offer new experimental perspectives (Decety and Cacioppo, 2012). According to the Social Relevance Hypothesis (SRH) (Neufeld et al., 2016), various capacities in social cognition crucially depend on social stimuli, to which a high degree of attentional relevance has been assigned automatically. Numerous social stimuli generate powerful bottom-up processes that produce automatic gestures. It is difficult to disregard, escape, or suppress such inputs in the social environment. In this context, the mu rhythm has been considered an oscillatory index of intentional action processing (Perry et al., 2011). Concretely, the mu rhythm has been suggested to play a crucial function in the sensorimotor transformation (Pineda, 2005) and the consciousness of motor action. For example, Simon and Mukamel (2016) studied the consciousness perception of hand movements displayed on videos with different degrees of visibility. Conscious perception was characterized by event-related desynchronization (ERD) of beta (15–25 Hz) oscillation approximately 500 ms after video onset, followed by mu (8–10 Hz) ERD oscillation at 800 ms. These ERDs were stronger in the contralateral sensorimotor cortex. During unconscious perception, only beta ERD occurred. These results are in favor of progressive recruitment of the neuronal activities of the mirror neuron system (MNS) from unconscious to conscious perception. The timing of the reported ERD (~500 ms) is compatible to the reentrant dynamic core concept (Edelman, 2003), which implies times for the activation of numerous loops integrating signals coming from the world, the body, and the self. The ability to be conscious of the actions and intentions of others has been suggested and supported by clinical studies, and is linked to the NMS (Avanzini et al., 2012; Neufeld et al., 2016). Patients with anosognosia induced by right frontal and parietal cortex lesions are not only unconscious of their own paralyzed limb, but also unconscious of the same side limb of another person (Ramachandran and Rogers-Ramachandran, 1996). Mu ERD has been linked to the MNS, as it is reduced in the affected sensorimotor hemisphere in stroke patients observing a grasping hand movement (Frenkel-Toledo et al., 2014). However, its role as an index of the MNS seems to be compromised, and it would be related more to the sensory processing (Coll et al., 2017).

## The Individual Self and Body Identity

From the integrated information theory (Tononi et al., 2016), the complexity of the interconnected brain tissue quantifying the level of consciousness and movement consciousness originates from movement experienced together with the related and concomitant multi-sensory entries. The cause and effect relationships encoded during the movement experienced by the highly interconnected brain complex mechanisms will generate movement consciousness, which will be intrinsic and unified (Koch, 2018). Preservation of the individual self and body identity can be considered a premise for experiencing the consciousness of movement execution. Phase locking in beta oscillation has been shown in the superior temporal gyrus (BA39) in an experiment in which participants observed another person's hand movement, which triggered the electrical somatosensory stimulus they received (Cebolla et al., 2014). Self-aspects of experienced spatial unity would explain such involvement of the right angular gyrus (BA39) (Blanke et al., 2005). In the same experiment, alpha, beta, and gamma power spectrum increases were located in BA40 as part of the parietal operculum, which was explained as additional somatosensory information from the observer's body schema through reafferent signals associated with the observed action, which could be related to the preservation of his/ her individual “self” and “body identity” with respect to the person seated next to him performing the movement (Iacoboni et al., 1999).

## The Oscillations-Movement-Consciousness Triad

For Kleinschmidt et al. (2012) the perceptive consciousness results from a “handshake” between the representation of the properties of physical stimulus and endogenous perceptual inference. This inference is supported by both the sensory signals (bottom-up processing) and the semantic properties of the stimulus (top-down processing). Reporting of the conscious percept always implies the production of a movement and whatever the actuators used (finger, eye, or vocal cords).

Understanding the complex interrelationships in the oscillations-movement-consciousness triad may also be approached from the perspective of sleep research by using lucid dreaming as a paradigm (LaBerge et al., 2018). Lucid dreaming happens only during paradoxical sleep, which is characterized by suppression of body electromyography and H-reflex amplitude, reduced EEG alpha oscillation, and rapid eye movements (Jouvet, 1994). Intriguingly, experienced lucid dreamers can exercise volitional control over their dreamed action while dreaming. Concretely, the tracking of visually imaged traced signs by the dreamers' eyes, pursuing the dreamed images of the dreamers' thumbs, resulted in the corresponding shapes in the electrooculogram recordings (LaBerge et al., 2018).

The visual perceptive consciousness is strongly dependent on eye movements (Costela et al., 2017), which are unconsciously or consciously directed toward specific points of interest. This eye scanpath concept was initially examined by Noton and Stark (1971), and later extended to visual imagery (Brandt and Stark, 1997). Following these authors, being conscious of a picture in our environment is accompanied by a specific sequence of saccadic eye movements (scanpath) representing a “playing out of an internal control from sensory-motor representation of a picture in the brain.” The gaze control expressed by the head-eye scanpaths is central to visual saliency models (Henderson, 2017; Henderson and Hayes, 2018; Tanner and Itti, 2019), in which bottom-up, learned top-down, goal relevance, and knowledge-driven prediction features are integrated. An interesting and easily feasible experiment is the Rubin's picture, which demonstrates the oscillating nature of perceptive consciousness (Strüber and Stadler, 1999). The perception of the image can be either a vase or two opposed human face profiles (Parkkonen et al., 2008). The two perceptions continually and rhythmically alternate up to the point that one becomes dominant. As suggested by Leopold and Logothetis (1999), visual multi-stability is linked more to the expression of a behavior than to passive sensory responses. The mechanism may be assimilated to that of competitive and recurrent oscillations around a dynamic attractor, and basic models include excitatory and inhibitory neurons reciprocally interconnected, forming competitive structures and acting as dynamic attractors. This latter notion defended by Kelso (1995) and others (Başar-Eroglu et al., 1996; Kruse et al., 1996; Strüber and Stadler, 1999; Tognoli and Kelso, 2014) is in agreement with the theory of neuronal groups (Changeux, 1983; Edelman, 1987), who stated that the synchronization of the oscillating activity determines the establishment of coherently acting neuronal sets, leading to conscious perception of the world. However, both the oscillatory nature of brain function and the fundamental role of the ocular movements (Peterson and Eckstein, 2013) should be considered to gain further understanding of such perceptive consciousness. Fixating a central point in the field of view does not preclude the absence of movement. Recent evidence (Shelchkova et al., 2019) using high precision methods to record eye movements have extended the scanpath concept of active vision into a small high-acuity region of the visual field (<1°) during fixation periods. During this apparent immobility, jittery movements in the form of small saccades, microsaccades, and drifts keep the visual point of interest within the foveola. The gaze placement at certain points over the Rubin's picture determines the emergence of perceptive alternation (Engel et al., 2001). An unresolved question is whether such movement results from a voluntary conscious command. Similarly, body movement is driven ontogenetically by reaching a reward linked to an object or to a living being situated somewhere in space. For this, gaze movement is oriented precisely to the target by means of head-eye saccade commanded by the superior colliculus, which receives specific basal ganglia inputs depending on whether they are selected voluntary consciously or automatic subconsciously (Kim and Hikosaka, 2015). Interestingly, Parkkonen et al. (2008) showed that the perception of vase or face in the Rubin's picture is accompanied by a respective modulation of 12 and 15 Hz for magnetoencephalographic oscillations. Shen et al. (2019) demonstrated by means of EEG and intracranial recordings that variation in the intrinsic frequency peak of the alpha oscillation predicts the perceptive consciousness of the bistable Ternus display (He and Ooi, 1999).

All in all, we emphasized the hypothesis that movement is inescapable in understanding consciousness and that oscillatory brain activity is their essential mechanism. Before verifying this hypothesis, it will be necessary to fully understand the influence exerted by the default mode network during the resting state, the dialogue and the distinction of the bottom-up and top-down processes producing, respectively, unconscious and conscious movement, the influence of the social stimuli on such bottom-up and top-down processes, and the influence of the movement experience on the individual self. A fundamental role of the ocular movements should be reconsidered to gain further understanding of the perceptive consciousness and finally of the complex interrelationships in the oscillations-movement-consciousness triad.

## Author Contributions

All authors listed have made a substantial, direct and intellectual contribution to the work, and approved it for publication.

### Conflict of Interest Statement

The authors declare that the research was conducted in the absence of any commercial or financial relationships that could be construed as a potential conflict of interest.
